# Molecular Evaluation of the mRNA Expression of the *ERG11*, *ERG3*, *CgCDR1*, and *CgSNQ2* Genes Linked to Fluconazole Resistance in *Candida glabrata* in a Colombian Population

**DOI:** 10.3390/jof10070509

**Published:** 2024-07-22

**Authors:** Leidy Yurany Cárdenas Parra, Ana Elisa Rojas Rodríguez, Jorge Enrique Pérez Cárdenas, Juan Manuel Pérez-Agudelo

**Affiliations:** 1 Facultad de Ciencias para la Salud, Universidad de Caldas, Manizales 170004, Colombia; leidy.cardenas@ucaldas.edu.co (L.Y.C.P.); labmicro@ucaldas.edu.co (J.E.P.C.); juan.perez@ucaldas.edu.co (J.M.P.-A.); 2 Facultad de Ciencias de la Salud, Universidad Católica de Manizales, Manizales 170001, Colombia

**Keywords:** *Candida glabrata*, fungal drug resistance, reverse transcriptase polymerase chain reaction, fluconazole, molecular docking simulation, multivariate analysis

## Abstract

Introduction: The study of *Candida glabrata* genes associated with fluconazole resistance, from a molecular perspective, increases the understanding of the phenomenon with a view to its clinical applicability. Objective: We sought to establish the predictive molecular profile of fluconazole resistance in *Candida glabrata* by analyzing the *ERG11*, *ERG3*, *CgCDR1*, and *CgSNQ2* genes. Method: Expression was quantified using RT-qPCR. Metrics were obtained through molecular docking and Fisher discriminant functions. Additionally, a predictive classification was made against the susceptibility of *C. glabrata* to fluconazole. Results: The relative expression of the *ERG3*, *CgCDR1*, and *CgSNQ2* genes was higher in the fluconazole-resistant strains than in the fluconazole-susceptible, dose-dependent strains. The gene with the highest relative expression in the fluconazole-exposed strains was *CgCDR1*, and in both the resistant and susceptible, dose-dependent strains exposed to fluconazole, this was also the case. The molecular docking model generated a median number of contacts between fluconazole and *ERG11* that was lower than the median number of contacts between fluconazole and *ERG3*, -*CgCDR1*, and -*CgSNQ2*. The predicted classification through the multivariate model for fluconazole susceptibility achieved an accuracy of 73.5%. Conclusion: The resistant strains had significant expression levels of genes encoding efflux pumps and the *ERG3* gene. Molecular analysis makes the identification of a low affinity between fluconazole and its pharmacological target possible, which may explain the lower intrinsic susceptibility of the fungus to fluconazole.

## 1. Introduction

*Candida* spp. is an etiological agent frequently isolated in infections, particularly, those caused by *Candida glabrata,* which are crucial, and so evidence must be collected to increase the understanding of the molecular implications, as well as an appropriate therapeutic approach, given the increasing incidence of such infections, especially in immunocompromised patients and its ability to develop resistance to or its susceptibility to being dose-dependent on a group of commonly used antifungals [1,2,3,4]. Studying the molecular mechanisms underlying drug resistance is essential for designing more effective therapeutic strategies and for anticipating and properly managing infections by this yeast in clinical settings. The scientific literature has documented various genetic and epigenetic factors that contribute to azole resistance in *C. glabrata*, providing details that can be considered for various research purposes [1,2].

In Colombia, *C. glabrata* is a pathogen that significantly contributes to the burden of fungal diseases. Epidemiological studies have shown that the prevalence of *C. glabrata* is increasing, particularly in clinical and hospital settings [3,4,5,6,7]. The frequency of isolation and the resistance or dose-dependent susceptibility exhibited by *C. glabrata* to azoles represent a significant therapeutic challenge and underline the importance of studies aimed at improving therapeutic effectiveness, as well as the continuous surveillance of antifungal resistance in the country.

Resistance can be caused by multiple factors, for example, frequent exposure of the fungus to antifungals, where its ability to evolve and adapt to hostile environments ensures its survival. Likewise, the exposure of the biological agent to conventional drug action mechanisms and the lack of new antifungal active principles with alternative mechanisms of action make treatment complex due to resistance, which is due to the structural and functional biological adaptive adjustments of the fungi, resulting in decreased pharmacological effectiveness [8,9].

Typically, azoles act by blocking lanosterol 14-alpha-demethylase, a cytochrome P450 enzyme that converts lanosterol, a structural intermediate of the fungal membrane, into ergosterol. From a molecular perspective, the free nitrogen atom located in the azole ring of fluconazole binds to the iron atom of the heme group of lanosterol 14-alpha-demethylase, disrupting the enzyme’s catalytic activity and, thus, interrupting the formation of the fungal membrane [10].

A resistance-associated mechanism involves the drug’s extrusion from inside the fungal cell, in which proteins or pumps belonging to two superfamilies, ATP Binding Cassette and MFS, are involved, and they are encoded by the *CgCDR1*, *CgSNQ2*, *PDH1*, and *MDR* genes [11,12,13,14,15,16,17,18,19,20,21]. Another resistance mechanism is associated with alterations in expression and mutations of the genes encoding the enzymes involved in the ergosterol biosynthesis pathway, such as lanosterol 14-alpha-demethylase and C-5 desaturase, which are responsible for synthesizing toxic sterols that accumulate in a fungus when it is exposed to azoles.

The *ERG11* gene encodes the lanosterol 14-alpha-demethylase enzyme, and its overexpression and/or mutations are associated with resistance, and this biological basis responds to maintaining the integrity of the fungal membrane in the presence of azoles and decreasing the affinity between the azole and the enzyme acting as the pharmacological target. On the other hand, mutations in the *ERG3* gene, which encodes the C-5 desaturase enzyme, have been described as resistance-associated mechanisms, as the enzyme loses activity and stops synthesizing toxic sterols in the presence of azoles, facilitating the survival of a fungus. Moreover, pathways for the synthesis of constitutive compounds of the fungal membrane have been found to be activated [22,23,24,25]. These genes have been identified in previous studies as important determinants of azole resistance in *C. glabrata*, making them ideal for molecular research aimed at understanding its resistance mechanisms [11,23,26].

Overall, the described mechanisms allow this fungus to avoid or reduce the effectiveness of azoles, a situation that necessitates further research to improve the understanding of this phenomenon at a local level and from a molecular perspective to make the problem visible in order to advance a comprehensive approach. In this study, the expression of the *ERG3*, *ERG11*, *CgSNQ2*, and *CgCDR1* genes of *C. glabrata* was evaluated using reverse transcriptase polymerase chain reaction (RT-qPCR), and high-level computational and data analytics routes were involved in contributing to the understanding and explanation of the phenomenon.

## 2. Materials and Methods

### 2.1. Study Type

A general experimental approach was carried out, contributing to the explanatory level of the research.

### 2.2. Strains and Sample-Handling Conditions

Sixteen strains of *C. glabrata*, collected in a previous study conducted in an intensive care unit in the city of Manizales by the Biosalud research group at the Universidad de Caldas, were used. Species confirmation of these strains was performed using the Vitek 2 compact system (Biomerieux, Marcy-l’Étoile, France), and their viability was established by adequate growth in culture media such as Sabouraud (Scharlau Microbiology, Sentmenat, Spain) and potato dextrose agar (PDA, Scharlau Microbiology, Sentmenat, Spain).

### 2.3. Antifungal Susceptibility Testing

Each strain underwent antifungal susceptibility testing in accordance with the protocols proposed by the Clinical and Laboratory Standards Institute (CLSI) version M27-A4 [27]. *Candida* isolates were grown on Sabouraud dextrose agar (Scharlau, Microbiology, Sentmenat, Spain) at 37 °C for 48 h. Antifungal susceptibility testing was performed in 96-well plates. Briefly, colonies of *C. glabrata* were re-suspended in RPMI 1640 medium (MP Biomedicals, LLC, Santa Ana, CA, USA), resulting in final concentrations of 0.5 × 10^3^ to 2.5 × 10^3^ cells/mL in the inoculum. Samples in the microplates were incubated at 37 °C for 48 h, and the minimum inhibitory concentrations (MICs) were evaluated spectrophotometrically at A 600 using a microplate reader. The antifungal drug tested in this study was fluconazole, which was dissolved and diluted according to CLSI recommendations [27]. MIC endpoints were assigned as the lowest concentration of antifungal drug that resulted in 50% growth inhibition compared with the drug-free control well for fluconazole. *Candida albicans* ATCC 90028 and *Candida glabrata* ATCC 90030 were used as the quality control organisms [27].

The MIC endpoint was used to interpret the resistance breakpoint values of the antifungal drugs according to CLSI guidelines and subsequent reports [28,29]. The MIC breakpoint values are as follows: resistant MIC > 64 µg/mL and SDD MIC ≤ 32 µg/mL.

### 2.4. Fluconazole Exposure Test

The fluconazole exposure assay was developed using liquid Sabouraud medium (Scharlau, Microbiology, Sentmenat, Spain) as the culture medium, and the established concentrations of the antifungal were used according to the protocol proposed by the CLSI broth dilution method. All tubes were incubated at 35 °C until the logarithmic phase of yeast growth occurred. The sediment size in each tube containing fluconazole (test group: exposed to fluconazole) was compared with the positive control (control group: not exposed to fluconazole). Cells were collected from both the positive control tube and the fluconazole tube, where cell growth was adjusted to a concentration between 2 × 10^8^ and 3 × 10^8^ yeast/mL. The antifungal concentration of the collected samples exposed to fluconazole is shown in Table 1.

The tubes selected in the antifungal exposure test were centrifuged to obtain yeast sediment, which was stored in DNase- and RNase-free Eppendorf tubes, and RNAlater (Invitrogen™: Thermo Fisher Scientific, Waltham, MA, USA) was later added to preserve them at −80 °C until extraction.

### 2.5. RNA Extraction

In the gene expression experiments performed, the guidelines proposed in the Minimum Information for Publication of Quantitative Real-Time PCR Experiments (MIQE) were followed. For RNA extraction, the protocol suggested in the extraction kit used (RiboPure–Yeast Kit, Thermo Fisher Scientific, Waltham, MA, USA) was followed according to the manufacturer’s recommendations. 

### 2.6. Quality Criteria for RNA Extraction 

The RNA solution was subjected to 1% agarose gel electrophoresis to observe the integrity of the genetic material. The purity of the RNA was measured using a UVIS Drop UVS99™ spectrophotometer (Thermo Fisher Scientific, Waltham, MA, USA) with readings of sample absorbance at 230, 260, and 280 nm. RNA purity values with a ratio of 260:280 and 260:230 greater than or equal to 2 were considered high purity and subsequently used for the RT-qPCR.

To evaluate the presence of DNA after treatment with DNAase I, PCR amplification of one of the RNA samples, without reverse transcription and in the presence of a universal primer for *C. glabrata*, was performed to assess if there was amplification of residual DNA. The DNA-contaminated strains underwent a new DNAase I treatment.

### 2.7. Protocol Standardization

For standardization, the *C. glabrata* ATCC 90030 strain was used as a reference for a susceptible dose-dependent strain, and a strain from the fluconazole-resistant group was randomly chosen. All procedures were performed according to previously established guidelines to ensure adequate reaction purity and to prevent RNA contamination by other microorganisms.

### 2.8. Reverse Transcription (RT) Protocol

The reverse transcription process was performed using a StepOnePlus Real-Time PCR System thermocycler (Applied Biosystems, Waltham, MA, USA) with the following protocol: incubation at 25 °C for 10 min and again at 42 °C for 15 min, and then the enzyme was inactivated at 85 °C for 5 min. The cDNA was converted from 1 µg of purified RNA using a SensiFastTM cDNA kit (Bioline, London, UK). The manufacturer’s instructions were meticulously followed without making any modifications to the protocol.

### 2.9. Selection and Optimization of the Primers for the qPCR

The primers for the reaction, both for the endogenous reference control gene and the resistance genes (*ERG3*, *ERG11*, *CgSNQ2*, and *CgCDR1*), are shown in Table 2.

### 2.10. qPCR Experiment

Each of the sample assays with and without fluconazole exposure was performed with identical triple replicates on a StepOnePlus Real-Time PCR system using a PowerUp SYBR Green Master Mix kit (Applied Biosystems, Waltham, MA, USA). The specificity of the qPCR reaction products was evaluated by analyzing the dissociation curves (melting curve).

Each qPCR quantification reaction contained PowerUp SYBR Green Master Mix [2X], 0.8 μM of each primer pair, and 10 ng/μL of cDNA. The program consisted of an initial denaturation in the following 2 steps: 50 °C for 2 min and 95 °C for 10 min, followed by 40 cycles of denaturation at 95 °C for 15 s and extension at 60 °C for 1 min. In order to establish the cDNA dissociation temperature and identify the non-specific amplification reactions, the following parameters were used: 95 °C for 15 s, 60 °C for 1 min, and 95 °C for 15 s.

### 2.11. qPCR Reaction Analysis 

The ΔCt comparative analysis method was used to establish the differences between the threshold cycles (Ct) of the resistance genes (*ERG11*, *ERG3*, *CgCDR1*, and *CgSNQ2*) and the endogenous reference control gene (*CgURA3*). The analysis of the results was performed using StepOnePlus Real-Time PCR™ software version 2.3 (Applied Biosystems, Waltham, MA, USA). This software also made the evaluation of the efficiency of each reaction possible.

### 2.12. Molecular Docking

The molecular docking procedure was carried out using the amino acid sequence and the predicted tertiary structures of the *ERG3*, *ERG11*, *CgCDR1*, and *CgSNQ2* proteins (Uniprot codes P50860, P50859, Q6FK23, and Q6FQN3, respectively) of *C. glabrata*. The maximum resolution of the available crystal structures corresponded to 2.0 Å. Protein models were downloaded from the online biological database CANDIDA-GENOME DATABASE, with the structural predictions coupled to the ALPHAFOLD program version 2.3.25 [33,34]. The FASTA format was considered preliminarily for the sequence handling for each protein. The nomenclature example for *ERG3* corresponded to the following: >CAGL0F01793g|ERG3 COORDS: ChrF_C_glabrata_CBS138: 201866-200772C,MDLVLETLDHYIFDDVYAKIAPVELQRGIDDSLVNALSLNKIVSNSTLLHETLSITNSLKRVNKDVYGLTPFLFDFTEKTYASLLPRNNLIREFFSLWAVVTVFGLLLYLITASLSYVFVFDRTIFNHPKYLKNQMYLEIKLAVSAIPTMSLLTVPWFMLELNGYSKLYYDVDWEHHGLRKLLIEYATFIFFTDCGIYLAHRWLHWPRVYKALHKPHHKWLVCTPFASHAFHPVDGYFQSLSYHIYPMILPLHKISYLILFTFVNFWSVMIHDGQHMSNNPVVNGTACHTVHHLYFNYNYGQFTTLWDRLGGSYRRPEDSLFDPKLKMDKKVLEKQARETAAYIQEVEGDDTDRVYNTDKKKTN.

InterProScan Analysis (Ref) confirmed the membership of the sequences with the protein families associated with the fluconazole resistance genes in *C. glabrata*. [35].

A structural and geometric suitability evaluation of the proteins of interest was executed using the SWISSMODEL-STRUCTURE ASSESSMENT (https://swissmodel.expasy.org/, accessed on 1 November 2023) platform. Metrics from Ramachandran plots (favorable angles) of > 96% and the MolProbity Score estimator of <1.0 were utilized [36].

The optimized formats .mol2 for fluconazole and .pdb for the three-dimensional protein structures of interest were used. The analysis generated by the AVOGADRO program version 1.2.0 was used for the energy optimization of the substrate (fluconazole) [37]. The exploratory docking process was developed using the open-source programs PyRx-AutoDock Vina version 0.8 and CHIMERA version 1.16, with a grid box size of 26 Å × 26 Å × 26 Å and the protein-loading and ligand files in .pdbqt extensions [38]. The molecular result metrics are presented with Delta-Gibbs values or in Gibbs free energy (ΔG kcal/mol).

### 2.13. Statistical Analysis

According to the normality tests of the relative expression results of each gene of interest, parametric (Student’s t) or non-parametric (Wilcoxon rank) statistics were used to determine differences in the means or medians. An alpha (α) of 0.05 was established as the level of statistical significance.

The multivariate analysis was based on the Fisher’s linear discriminant functions route to provide a predictive classification score against the susceptibility (resistant or susceptible and dose-dependent) of *C. glabrata* [39,40]. The variables included in the multivariate model were as follows: gene, strain, mean RQ without fluconazole, and mean RQ with fluconazole.

The discriminant analysis was chosen as the proposed predictive model for the investigative process based on the intrinsic discriminant (dependence) capability sought in the research as follows: the classification capacity of the fluconazole susceptibility from the numerical independent variables, where the mean RQs with and without fluconazole and where the categorical options were the gene and strain, the confirmatory capacities of the new cases added to the model, obtaining the linear combination of independent variables that maximized the distinction of a group’s membership, the estimation or probability metric of classifying each particular case to a group of interest, and the absence of collinearity or multicollinearity among the independent variables [40].

This route suggested the generation of qualitative variables with numerical values associated with the categories (susceptible and dose-dependent = 1, resistant = 2; *ERG3* gene = 1, *CgCDR1* gene = 2, *CgSNQ2* gene = 3; strain 04-1A = 1, strain 07-1A = 2, strain 109-1A = 3, etc.). Numerical variables (the mean RQs with and without fluconazole) were assumed in their continuous values in the model. Statistical significance was considered with predictive model classification accuracies (discriminant function) of ≥ 70%. The biostatistical analysis was supported by the licensed IBM SPSS package version 26 [41].

## 3. Results

### 3.1. Analysis of the Relative Gene Expression 

As a result of the fluconazole susceptibility tests, the 16 strains were classified as follows: 6 were resistant (samples 06-3A, 95-1A, 116-5137-1184-1A, and 65-3) and 10 were susceptible and dose-dependent (samples 11-3A, 04-1A, 16-5A, 24-5A, 25-1A, 20-2A, 14-5A, 07-1A, 68-4, and 109-1A). For the analysis of the relative expression results of the *CgCDR1*, *ERG11*, *ERG3*, and *CgSNQ2* genes, the value of the relative expression of the *CgURA3* gene, corresponding to 1, was used as a reference. In other words, relative expression results greater than 1 were considered to be overexpressed. Additionally, through parametric and non-parametric analysis routes, the medians and/or averages of the relative expression levels of genes based on exposure or non-exposure to fluconazole were compared to determine the differences in the expression levels.

**Figure 1:**
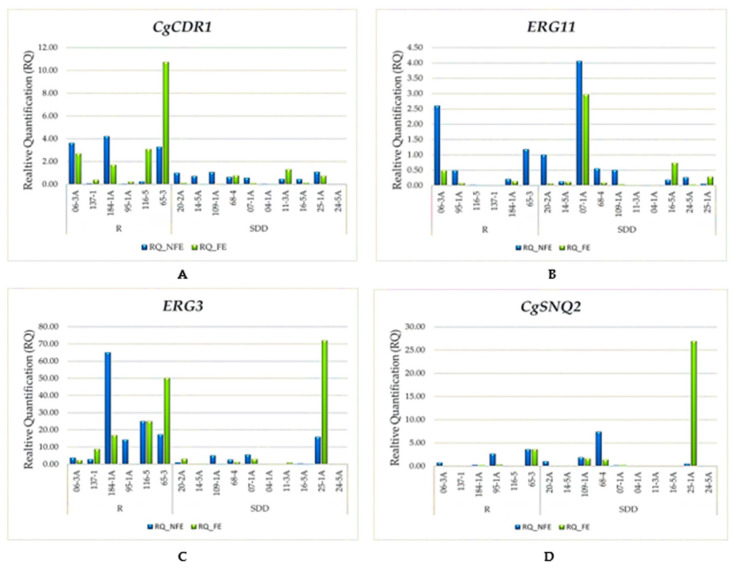
(**A**) Relative expression levels of the *CgCDR1* gene for strains not exposed vs. strains exposed to fluconazole. (**B**) Relative expression levels of the *ERG11* gene for strains not exposed vs. strains exposed to fluconazole. (**C**) Relative expression levels of the *ERG3* gene for strains not exposed vs. strains exposed to fluconazole. (**D**) Relative expression levels of the *CgSNQ2* gene for strains not exposed vs. strains exposed to fluconazole.

As observed in Figure 1, the relative expression (RQ) levels of the studied genes in the 16 strains, under the condition of exposure and non-exposure to fluconazole, regardless of the antifungal susceptibility profile, indicated that the relative expression levels of the exposed strains were higher than those of the non-exposed strains in *ERG3* (the difference in the RQs between the exposed and non-exposed strains was 1.61, with *p* = 0.776), followed by *CgSNQ2* (the difference in the RQs between the exposed strains and the non-exposed strains was 1, with *p* = 0.044), and, lastly, *CgCDR1* (the difference in the RQs between the exposed strains and the non-exposed strains was 0.3, with *p* = 0.523). In contrast, the *ERG11* gene in the strains exposed to fluconazole showed higher relative expression levels than the non-exposed strains (the difference in the RQs between the exposed strains and the non-exposed strains was −0.38, with *p* = 0.034).

Of the six strains classified as resistant to fluconazole, regardless of exposure to the antifungal, all overexpressed at least one gene, and it is noteworthy that the *ERG3* gene was overexpressed in all of them, the *CgCDR1* gene was overexpressed in four (06-3A, 184-1A, 116-5, and 65-3), the *ERG11* gene was overexpressed in two (06-3A and 65-3), and the *CgSNQ2* gene was overexpressed in two (95-1A and 65-3).

Strain 65-3 had higher relative expression of the *CgCDR1* and *ERG3* genes compared to the other strains, contrasting with a very low relative expression of *ERG11*. Additionally, under the condition of no exposure to fluconazole, it overexpressed all four genes studied, while the same strain exposed to the antifungal did not overexpress the *ERG11* gene. A similar phenomenon occurred with strain 06-3A, which, without being exposed, overexpressed the *CgCDR1*, *ERG11*, and *ERG3* genes, and when exposed to the antifungal, it did not overexpress the *ERG11* gene.

The descriptive analysis and the results of the parametric and non-parametric tests on the relative expression levels of the genes studied are reported in Table 3.

**Figure 2:**
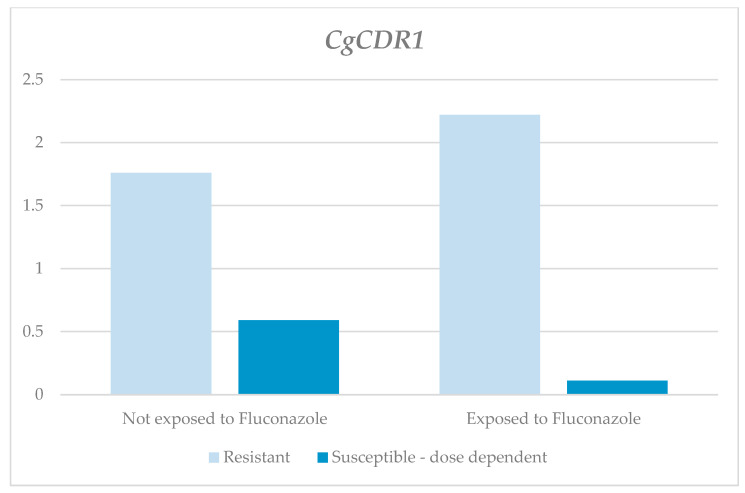
Relative expression of the *CgCDR1* gene according to susceptibility profile and the exposure to fluconazole.

As shown in Table 1 and Figure 2, the relative expression levels of the *CgCDR1* gene in the fluconazole-exposed, resistant strains were higher than those in the non-exposed ones. However, these differences were not statistically significant (exposed, 2.22; non-exposed, 1.76; *p* = 0.231). On the contrary, the fluconazole-exposed, susceptible, dose-dependent strains were found to have lower relative expression levels than the non-exposed ones, and a statistically significant difference (RQ value for the susceptible, dose-dependent strains that were exposed was 0.11, and for the non-exposed, it was 0.59, with *p* = 0.046) was identified. According to the classification based on the susceptibility profile, higher RQ medians were obtained for the resistant strains, both for those exposed and those not exposed to fluconazole, compared to the susceptible, dose-dependent ones.

**Figure 3:**
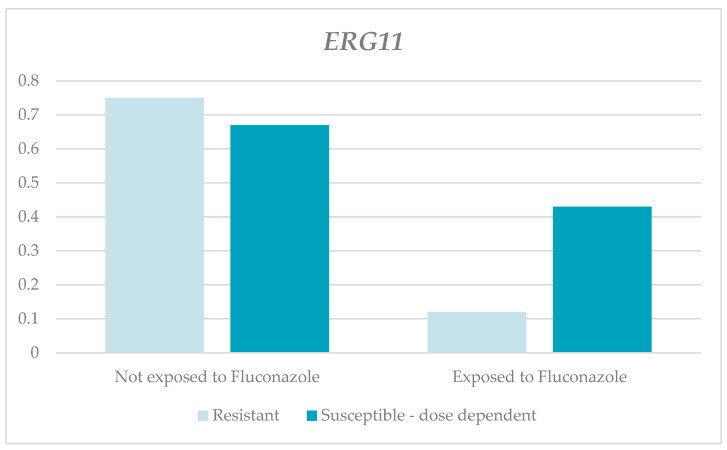
Relative expression of the *ERG11* gene according to susceptibility profile and the exposure to fluconazole.

The relative expression levels of the *ERG11* gene in the resistant and susceptible, dose-dependent strains were lower in those exposed to fluconazole compared to those not exposed. Nevertheless, these differences were not significant (RQ-resistant for those exposed, 0.12, and for those not exposed, 0.75, with *p* = 0.065; RQ-susceptible and dose-dependent for those exposed, 0.43, and for those not exposed, 0.67, with *p* = 0.086). Between the two groups and according to the susceptibility profiles, there were higher average RQs in the susceptible, dose dependent strains than in the resistant ones (Table 1 and Figure 3).

**Figure 4:**
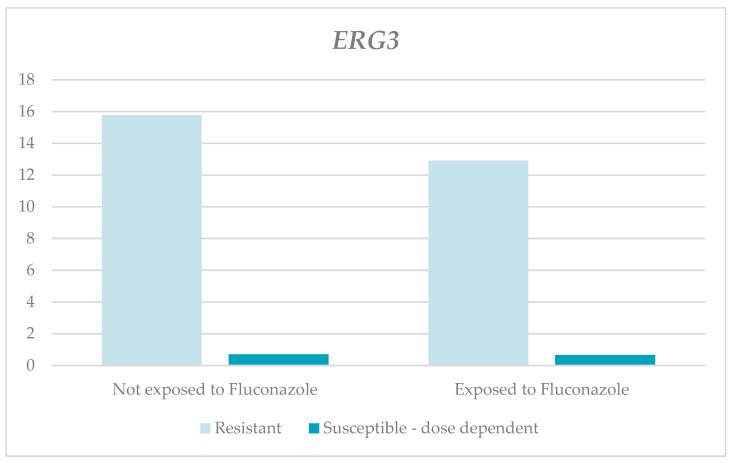
Relative expression of the *ERG3* gene according to susceptibility profile and the exposure to fluconazole.

On the other hand, the relative expression levels of the *ERG3* gene in the resistant and susceptible, dose-dependent strains exposed to fluconazole were lower than in those not exposed, with statistically non-significant differences (RQ-resistant for those exposed, 12.9, and for those not exposed, 15.77, with *p* = 0.343; RQ-susceptible and dose-dependent for those exposed, 0.66, and for those not exposed, 0.71, with *p* = 0.399). There were higher RQ medians in the resistant strains, for both those exposed and those not exposed to fluconazole, than in the susceptible, dose-dependent strains, as can be observed in Table 1 and Figure 4.

**Figure 5:**
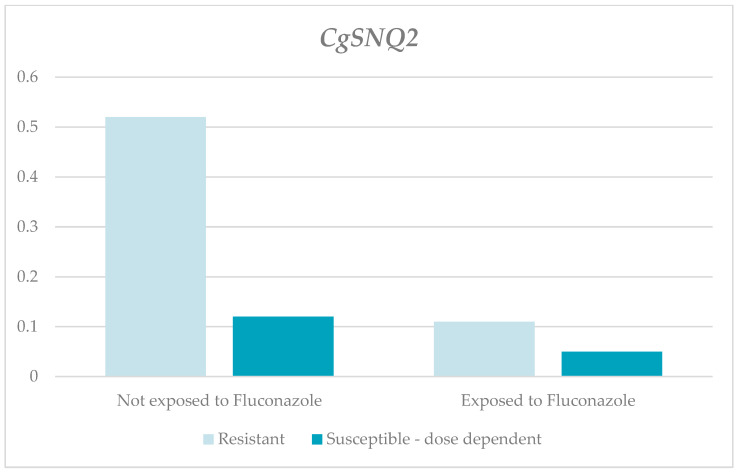
Relative expression of the *CgSNQ2* gene according to susceptibility profile and the exposure to fluconazole.

Lastly, the relative expression levels of the *CgSNQ2* gene in both the resistant and susceptible, dose-dependent strains were lower in the strains exposed to the antifungal. However, the differences were only significant for the resistant group (RQ-resistant for those exposed, 0.11, and for those not exposed, 0.52, with *p* = 0.014; RQ-susceptible and dose-dependent for those exposed, 0.05, and for those not exposed, 0.12, with *p* = 0.166). The RQ medians were higher in both the exposed and not-exposed resistant strains compared to the susceptible, dose-dependent strains (Table 1 and Figure 5).

### 3.2. Results of the Predictive Model on the Susceptibility Profile

The analysis model included four categorical and continuous predictor variables (gene, strain, RQ mean without fluconazole, and RQ mean with fluconazole) and one categorical predictor variable (fungus susceptibility profile). The multivariate evaluation through the route of the Fisher’s linear discriminant function or a dichotomous prediction (sum of the estimation products) generated the coefficients for the classification of susceptibility/susceptibility profile, as shown in Table 4.

The overall comparative classification results between the original susceptibility profile findings by the macrodilution tests as compared to the classifications predicted by the model yielded a classification accuracy of 73.5% (>0.7).

The utilization of the coefficient results is described below in Table 5. The findings in the research corresponded to the *CgCDR1* gene (categorical value = 2) from *C. glabrata* strain 07-1A (categorical value = 2), with an RQ mean without fluconazole of 0.57 and an RQ mean with fluconazole of 0.22. They were replaced with the product of the coefficients, and the products were added, as shown below.

### 3.3. Molecular Docking

The molecular processing using basic structural bioinformatic techniques (rigid docking) made obtaining the ΔG values for the relationship between the proteins expressed by the *C. glabrata* genes *ERG3*, *ERG11*, *CgCDR1*, and *CgSNQ2* and the fluconazole substrate possible. Table 6 describes the findings of the energetic molecular metrics for the main positions (interaction sites) of fluconazole and the mentioned proteins.

**Figure 6:**
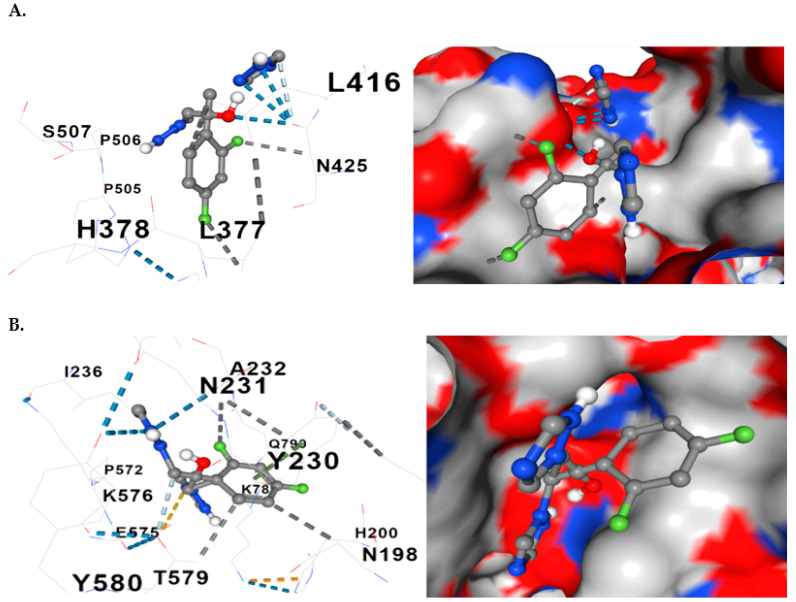
Comparative representations of fluconazole and the proteins of interest docking. (**A**) Three-dimensional presentation of the molecular relationship (docking) between fluconazole and *ERG11*. (**B**) Three-dimensional presentation of the molecular relationship between fluconazole and *CgCDR1*. Hydrophobic interactions with the residues are shown with dashed gray lines and with gray surfaces on the proteins.

The docking model also revealed the relationships between the constituent amino acids and fluconazole at the docking sites. Figure 6 shows two representative models of the process for fluconazole and *ERG11* and fluconazole and *CgCDR1.*

The results of the docking revealed a median of 10 detected contacts for the interaction between fluconazole and *ERG11*, and for the interactions between fluconazole and *CgCDR1*, *ERG3*, *and CgSNQ2*, there was a median of 13.

## 4. Discussion

This study addressed the quantification of gene expression in the species *C. glabrata*, encoding either the drug efflux transporters or the enzymes involved in the ergosterol biosynthesis pathway.

The genes encoding the addressed efflux transporters were *CgCDR1* and *CgSNQ2*, and their expression findings allowed us to identify that regardless of the fluconazole susceptibility profile, the strains exposed to the antifungal had higher relative expression levels than those not exposed, indicating a greater amount of pharmacological efflux transporters available for reducing the intracellular fluconazole concentrations in the experimental group treated with the antifungal. Moreover, it was possible to identify that the resistant strains also had higher expression levels than those classified as susceptible and dose-dependent.

Complementary findings from the molecular perspective showed that the detected phenotype of the *C. glabrata* activity established that there was a limited pharmacological response in the scenario of *CgCDR1* overexpression and that the affinity between fluconazole and *CgSNQ2* was superior to those of the other genes of interest in this study. Although no other studies using computational techniques for molecular docking were found, similar results were found in other studies where resistance to azoles was related to the overexpression of genes encoding efflux pumps, such as the *CgCDR1* and *CgSNQ2* genes related to the ATP-Binding Cassette (ABC) superfamily of proteins [11,14,23,42,43,44,45,46,47].

Researchers Karl, Joseph, and Tomas conducted a study on resistance in *C. albicans* and identified that the deletion of the *ERG11* gene was associated with the positive regulation of *ERG3* expression, as well as the presence of enzyme inhibitor drugs and genetic alterations in ergosterol biosynthesis, leading to increases in *ERG3* mRNA levels [48]. Although this finding does not correspond to the same species we used, it may behave biologically similarly to the *Candida* genus, since our study identified generally very low *ERG11* expression values, most of them even lower than one, which corresponded to the expression of the reference strain. In contrast, much higher *ERG3* expression values were evidenced.

Moreover, our results showed the overexpression of *ERG3* in almost all strains (both resistant and susceptible, dose-dependent), a situation that could be extrapolated to a lower susceptibility to the action of fluconazole. Nevertheless, *ERG3* expression stood out for its very high values in those classified as resistant compared to those classified as susceptible and dose-dependent. It is plausible that *ERG3* overexpression is an early cellular stress response to try to maintain membrane integrity in the presence of a blockade generated by azoles on an enzyme encoded by *ERG11*, particularly in *C. glabrata*. Being intrinsically less susceptible to fluconazole, this was a finding consistent with the results of molecular docking that would indicate a limited affinity between fluconazole and *ERG11* [49,50,51]. 

The available studies have focused on investigating loss-of-function mutations in *ERG3*, which may be biological responses to overexpression in which the inactivity of the enzyme encoded by *ERG3* resulting from the loss-of-function mutations leads to decreases in the synthesis of toxic intermediate sterols in the presence of azoles, favoring the survival of the fungus [14,18,20]. Nevertheless, these studies have not focused on quantifying expression, and so references that could provide valuable information about *ERG3* expression and azole/fluconazole resistance in *Candida*, especially in *C. glabrata*, are scarce, and the relationship between *ERG3* expression and azole resistance is not clear.

When the strains resistant to the antifungal were exposed, the expression of the *ERG11* gene decreased. The relative expression of the *ERG11* gene was low in both the resistant and susceptible, dose-dependent strains, as well as in the strains exposed to the antifungal compared to those not exposed, and only three strains showed expression levels above one. These results for *ERG11* gene expression may be related, as one of the biological conditions that explain the lower intrinsic susceptibility of *C. glabrata* to fluconazole compared to other *Candida* species could indicate that low gene expression may affect lanosterol 14-alpha-demethylase synthesis and decrease pharmacological efficacy by reducing the size of the pharmacological target [52]. 

There is limited scientific evidence on this subject, and the studies found do not report specific *ERG11* expression values in *C. glabrata*, making it necessary to propose more research to expand the conceptual framework.

This study presents several limitations. Firstly, 1% agarose gel electrophoresis was used to evaluate the RNA integrity, and this is a methodology considered to be limited in terms of identifying susceptibility and precision. It is important to note that there are currently more advanced and sensitive methodologies, such as the bioanalyzer, which allow for a more exhaustive and quantitative evaluation of RNA quality. Nonetheless, this technology could not be used due to a lack of technological availability in the region. Additionally, the scope of the study did not allow for the evaluation of other molecular mechanisms, such as point mutations in the genes involved in ergosterol synthesis, due to financial constraints.

Despite the mentioned restrictions, the findings obtained provide a preliminary analysis of the probable role played by exposure to fluconazole in the development of resistance in *C. glabrata* isolates. These results support the recommendation to avoid unnecessary and inadequate prescription of this antifungal, as well as its prolonged use, and they emphasize the importance of basing its administration on accurate diagnosis confirmation and species susceptibility profiling.

## 5. Conclusions

The phenomenon of resistance is complex and depends on many factors inherent to this fungus, its host, and the antifungal drug used. It was possible to identify that the strains resistant to fluconazole had significant expression levels of genes encoding drug efflux pumps belonging to the ATP-Binding Cassette superfamily, and there was a much higher expression of the *ERG3* gene compared to the other genes involved in the analyses. This may be a particular characteristic of the *C. glabrata* species, with its comparatively low expression of the *ERG11* gene. These findings should motivate further research that favors a comprehensive understanding of the adaptation–resistance dynamics to antimicrobials.

To date, no similar work has attempted to understand this resistance phenomenon through the integration of categorical and numerical variables derived from molecular observations and fluconazole responses in *C. glabrata*. Therefore, this contribution may be important for the scientific community, and it may be taken as a reference for studies involving complementary routes that integrate the variables in predictive models that complement and streamline such analyses. In addition, this type of molecular analysis allows for integrating an explanatory scenario of the relationship between widely used antimicrobials and the biological activity represented by *C. glabrata*. Considering that such studies can help scientific and clinical communities better understand the biological basis of resistance, they may contribute key elements to solving this problem with predictive, applicative, and therapeutic precision.

## Figures and Tables

**Table 1:** Fluconazole concentrations of the collected samples exposed to the antifungal.

Sample	Fluconazole Concentration
06-3A	8 µg/mL
95-1A	4 µg/mL
116-5	8 µg/mL
137-1	4 µg/mL
184-1A	8 µg/mL
65-3	16 µg/mL
11-3A	16 µg/mL
04-1A	32 µg/mL
16-5A	8 µg/mL
24-5A	8 µg/mL
25-1A	16 µg/mL
20-2A	16 µg/mL
14-5A	32 µg/mL
07-1A	8 µg/mL
68-4	8 µg/mL
109-1A	8 µg/mL

**Table 2:** Primer Sequences.

Gene		Primer Sequence (5′–3′)	Reference
*CgCDR1*	Forward	CATACAAGAAACACCAAAGTCGGT	[30]
	Reverse	GAGACACGCTAACGTTCACCAC	
*ERG11*	Forward	TCGGTCCATCTCTGTTTCTT	[31]
	Reverse	GAACACTGGGGTGGTCAAGT	
*ERG 3*	Forward	AAGCGTGTGAACAAGGAC	[30]
	Reverse	GCGTAGGTCTTCTCTGTGA	
*CgSNQ2*	Forward	CGTCCTATGTCTTCCTTACACCATT	[30]
	Reverse	TTTGAACCGCTTTTGTCTCTGA	
*CgURA3*	Forward	GAAAACCAATCTTTGTGCTTCTCT	[32]
	Reverse	CATGAGTCTTAAGCAAGCAAATGT	

**Table 3:** Relative expression values of genes *CgCDR1*, *ERG11*, *ERG3*, and *CgSNQ2* according to susceptibility profile and exposure to the antifungal.

Analyzed Gene	Exposure to the Antifungal	Resistant One-Tailed Analysis						Susceptible and Dose-Dependent One-Tailed Analysis					
		Mean	Median	Minimum	Maximum	DE	*p*	Mean	Median	Minimum	Maximum	DE	*p*
*CgCDR1*	Without Fluconazole	1.91	1.76	0.05	4.22	1.99	0.231 ^1^	0.6	0.59	0.04	1.09	0.38	0.046 ^1^
	With Fluconazole	3.15	2.22	0.24	10.75	3.89		0.33	0.11	0.01	1.33	0.45	
*ERG11*	Without fluconazole	0.75	0.35	0.01	2.6	1.00	0.065 ^2^	0.67	0.22	0	4.06	1.23	0.086 ^2^
	With fluconazole	0.12	0.04	0.01	0.49	0.18		0.43	0.08	0	2.97	0.91	
*ERG3*	Without fluconazole	21.39	15.77	2.93	65	22.9	0.343 ^1^	3.09	0.71	0	15.9	4.97	0.399 ^1^
	With fluconazole	17.28	12.94	0.01	50.3	18.64		8.1	0.66	0.01	72.12	22.51	
*CgSNQ2*	Without fluconazole	1.22	0.52	0.01	3.59	1.53	0.014 ^1^	1.1	0.12	0	7.4	2.29	0.166 ^1^
	With fluconazole	0.69	0.11	0	3.59	1.42		3.03	0.05	0	26.95	8.42	

^1^, One-tailed Wilcoxon rank-sum test; ^2^, one-tailed Student’s *t*-test.

**Table 4:** Coefficients in the classification function for the susceptible dose-dependent (SDD) or resistant profiles.

	General Susceptibility Profile	
Variables in the Model	Resistant	SDD
Gene analyzed	4.141	3.730
Categorized strain	0.369	0.276
RQ mean without fluconazole	0.363	0.302
RQ mean with fluconazole	0.111	0.042
Constant	−8.009	−5.820

**Table 5:** Use of the results of the model coefficients.

	Susceptibility Profile		Forecasted Profile	
Variables in the Model	Resistant	SDD	Resistant	SDD
Gene analyzed	4.141	3.730	2 × 4.141	2 × 3.730
Categorized strain	0.369	0.276	2 × 0.369	2 × 0.276
RQ mean without fluconazole	0.363	0.302	0.57 × 0.363	0.57 × 0.302
RQ mean with fluconazole	0.111	0.042	0.22 × 0.111	0.22 × 0.042
Constant	−8.009	−5.820	1 × (−8.009)	1 × (−5.820)
		Total	1.2425	2.3738

The total values were compared, and the highest value defined the forecasted profile. In this case, under the example’s characteristics, the SDD category was forecasted. This classification was considered correct (accurate) by the model in 73.5% of the cases.

**Table 6:** Representation of the ΔG (kcal/mol) values for the various positions generated by the docking model for fluconazole and the proteins of interest in *C. glabrata*.

		Fluconazole	
Protein	Model	ΔG (Kcal/mol)	Number of Contacts
*ERG3*	1.	−6.2	19
	2.	−6.1	13
	3.	−6.0	6
	4.	−5.9	11
	5.	−5.8	6
*ERG11*	1.	−2.8	12
	2.	−2.6	11
	3.	−2.5	11
	4.	−2.4	10
	5.	−2.2	9
*CgCDR1*	1.	−7.0	14
	2.	−6.4	14
	3.	−6.4	15
	4.	−6.2	14
	5.	−6.2	11
*CgSNQ2*	1.	−7.6	17
	2.	−7.5	12
	3.	−7.4	12
	4.	−7.2	11
	5.	−7.1	11

## Data Availability

The original contributions presented in the study are included in the article/Supplementary Materials, further inquiries can be directed to the corresponding author.

## Supplementary Materials

The following supporting information can be downloaded at https://www.mdpi.com/article/10.3390/jof10070509/s1. Raw data: *Candida glabrata* Database.
